# Response to retraction request and allegations of misconduct and scientific flaws

**DOI:** 10.2807/1560-7917.ES.2021.26.5.2102041

**Published:** 2021-02-04

**Authors:** Eurosurveillance editorial team

**Affiliations:** 1 European Centre for Disease Prevention and Control (ECDC), Stockholm, Sweden

On 27 November 2020, the *Eurosurveillance* editorial office was made aware of concerns and a request to retract the article ‘Detection of 2019 novel coronavirus (2019-nCoV) by real-time RT-PCR’ by Corman et al., published on 23 January 2020.

Allegations concerned the scientific quality of the article, the peer review process and a conflict of interest for two of the authors, who are also editorial board members of *Eurosurveillance*. The authors of the article and *Eurosurveillance*’s editorial board were informed about these claims on 2 December and 3 December 2020, respectively. On 3 December 2020, an editorial note was published stating that the *Eurosurveillance* editorial team would investigate the matter following the journal’s existing procedures and in consultation with experts [1]. The two co-authors of the article in question who are associate editors on *Eurosurveillance*’s editorial board were excluded from all related proceedings.

An initial evaluation of the allegations was undertaken in collaboration with several board members, followed by a discussion at the annual editorial board meeting on 4 December 2020. This resulted in the decision that it was not necessary to publish an expression of concern with respect to allegations of scientific misconduct or fraud, and that there was no conflict of interest by the associate editors who co-authored the manuscript. It was further decided to involve external subject experts to assess the allegations related to the scientific content of the article.

Members of the *Eurosurveillance* editorial board may submit manuscripts to the journal, in keeping with the policies of many other respected scholarly journals. However, articles submitted by members of the board are not given any priority over other manuscripts. When editorial board members are authors of a submitted manuscript, they are not involved in any stage of the peer review or the editorial decision-making, nor do they have access to confidential information related to the decision-making process. Whether or not authors are members of the editorial board has no bearing on editorial consideration, nor any effect on the editorial evaluation (https://www.eurosurveillance.org/evaluation) or review (https://www.eurosurveillance.org/for-reviewers) processes, which are implemented as described on the journal’s website. These principles were followed for the article by Corman et al., which was authored by 24 virologists and laboratory experts in six different countries; additionally, the editorial board of *Eurosurveillance* confirmed that when these principles are applied, being a board member does not constitute a conflict of interest. The journal has rejected articles submitted by board members on many occasions.

*Eurosurveillance* has a confidential peer review process and a policy of double-blind peer review, in which both the authors’ and the reviewers’ identities are confidential. Peer review reports are not made publicly available and are considered internal documents intended solely to guide editorial decision-making. Sharing reviewer reports with third parties would violate the assumed confidentiality obligations for journals that apply this peer review model, as per the International Committee of Medical Journal Editors [2] and the World Association of Medical Editors [3].

Following the allegations raised, all associate editors—except for the two co-authors of the article in question who are associate editors—were given access to the full documentation of the evaluation and review process the article underwent. This enabled them to confirm that the reviews were conducted in full and by independent experts.

The article by Corman et al. was fast-tracked and was reviewed by two independent experts in the field. The editorial team decided that this submission warranted quick consideration in the context of an exceptional and rapidly evolving public health situation and a need to enable laboratories to detect the newly emerged virus. When the article was submitted, the situation in Hubei province, China, had evolved massively. Several cases of what was later named coronavirus disease (COVID-19) had been imported to other countries and China had taken unprecedented, large-scale measures to stop virus spread. Thus, after a pre-submission enquiry—a common practice for scholarly journals—we contacted potential peer reviewers who agreed to stand by to review the submission should it pass the initial screening conducted by the editorial team. The reviewers kindly picked up their assignments immediately and submitted their overall positive verdicts hours after submission. The authors were also asked to stand by, as the editors would need them to be available on short notice to reply to the reviewers’ comments and to respond to editorial queries and comments. All parties worked intensely, including outside the usual working hours, on the finalisation of the submission.

The speed of the publishing process has led to allegations via social media and email that the evaluation and review process were flawed. The *Eurosurveillance* editorial team has long-standing experience in expedited publishing in instances when rapid dissemination of information could potentially lead to a prompt change in an ongoing public health situation or create awareness for topics of timely relevance. In such instances, the editorial team works in close coordination with reviewers and authors. Since 2015, about 30% of rapid communications have been published less than 2 weeks following submission, including peer review. This has also been the case for a maximum of two regular articles per year. *Eurosurveillance*’s in-house editorial team performs most editorial and all publishing tasks, without involvement of external parties such as typesetters. This allows for great flexibility, particularly in times of emerging or evolving public health emergencies [4-6], when case numbers or other relevant information/data can be updated even hours before publication.

Expedited review does not necessarily affect the filtering function of peer review, nor does it compromise reviewers’ ability to critically assess the content, validity and quality of a paper. The article by Corman et al. is a methodological paper describing the setup and validation of a PCR-based workflow that would enable laboratories to detect the then novel severe acute respiratory syndrome coronavirus 2 (SARS-CoV-2). As pointed out in an accompanying editorial note on 23 January 2020 [7], the primers and probes and a brief description had already been made available in the form of a World Health Organization Interim Guidance document [8], while the article further elaborated on the details of the validation in five different international laboratories. 

Of note, the 23 January 2020 issue also contained an article by Wu et al. that assessed the epidemiological characteristics of COVID-19 infections [9], for which we were able to secure an equally fast peer review.

The detailed allegations with respect to scientific flaws in the Corman et al. article were reviewed by a group of five laboratory experts. These comments were made available to the *Eurosurveillance* associate editors, except for those who were co-authors of the paper.

The consulted experts confirmed that the Corman et al. article was scientifically adequate for its purpose and for the limited data and material available at this early stage in the COVID-19 pandemic. Any laboratory deciding to use the primers and protocol suggested in this article would ascertain the assay for its fitness for purpose and compliance with local quality and accreditation requirements; this is what has happened worldwide since the publication of the article. With more data and evolving knowledge, laboratories have since further improved the initial method, as per usual practice.

In conclusion, after a thorough investigation in which we collected scientific advice from various sources, including several external reviewers, the editorial team—unanimously supported by its associate editors, except for those who were involved as co-authors—has decided that the criteria for a retraction of the article have not been fulfilled.
